# Acetyl-11-keto-β-boswellic acid attenuates titanium particle-induced osteogenic inhibition via activation of the GSK-3β/β-catenin signaling pathway

**DOI:** 10.7150/thno.35988

**Published:** 2019-09-23

**Authors:** Longbin Xiong, Yu Liu, Feng Zhu, Jiayi Lin, Dongxiang Wen, Zhen Wang, Jiaxiang Bai, Gaoran Ge, Congxin Xu, Ye Gu, Yaozeng Xu, Jun Zhou, Dechun Geng

**Affiliations:** 1 Department of Orthopaedics, The First Affiliated Hospital of Soochow University, Suzhou, 215006, China;; 2 Sun Yat-sen University Cancer Center; State key laboratory of Oncology in South China; Collaborative Innovation Center for Cancer Medicine, Guangzhou, Guangdong 510000, China;; 3 Department of Orthopaedics, Suzhou Kowloon Hospital Shanghai Jiao Tong University School of Medicine, Suzhou, Jiangsu 215006, China;; 4 Department of Orthopedics, Soochow University Affiliated First People's, Hospital of Changshou City, Changshu, China

**Keywords:** acetyl-11-keto-β-boswellic acid, peri-prosthetic osteolysis, titanium particle, bone formation, GSK-3β

## Abstract

**Rationale:** Peri-prosthetic osteolysis (PPO) is mainly induced by wear particles and represents the leading cause of implant failure and revision surgery. Previous studies have identified mitigation of wear particle-induced inflammation and bone resorption as the main approaches to treat PPO. Recently, wear particle-induced reduction of bone formation around the prosthesis was identified as a major factor in the development of PPO. Acetyl-11-keto-β-boswellic acid (AKBA), a derivative of frankincense, has been shown to play a potential role in bone metabolism. However, whether AKBA enhances bone formation in wear particle-induced osteolysis remains unknown. In this study, we examined whether AKBA attenuates titanium particle-induced osteogenic reduction.

**Methods:** Titanium particles were used to induce osteolysis in murine calvaria, and micro-CT and histological analyses were used to evaluate the results. Mouse osteoblast cells, MC3T3-E1 were co-cultured with titanium particles to determine their effect on osteoblast formation *in vitro*.

**Results:** We demonstrated that AKBA treatment significantly inhibited titanium particle-induced osteogenic inhibition by enhancing osteogenesis both *in vivo* and *in vitro*. AKBA treatment also enhanced the phosphorylation of GSK-3β, decreased the degradation of β-catenin, and increased the translocation of β-catenin from the cytoplasm to the nucleus. Taken together, these results showed that AKBA treatment attenuated titanium-induced osteogenic inhibition by activating the GSK-3β/β-catenin signaling pathway.

**Conclusion:** These findings suggest that AKBA is a promising new target in the prevention and treatment of PPO.

## Introduction

Peri-prosthetic osteolysis (PPO) induced by wear particles is the primary cause of aseptic prosthesis loosening, which directly affects the service life of implants 1. More than three-quarters of total joint arthroplasty (TJA) implant loosening is due to aseptic osteolysis 2. Wear particles gather at the interface between the bone and implant, resulting in the stimulation of macrophages and fibroblasts and the secretion of inflammatory transmitters, such as tumor necrosis factor-α (TNF-α), prostaglandin E2, interleukin 1β (IL-1β), and IL-6 3. Secreted cytokines act on macrophages, osteoblasts, and osteoclasts and interfere with the RANKL/RANK/OPG signaling pathway, enhancing osteoclasts and weakening osteogenesis. The imbalance between osteoblastic bone formation and osteoclastic bone resorption ultimately leads to PPO 4-6. Osteoclasts have been recognized as a major factor underlying wear particle-induced osteolysis; however, many studies have recently identified wear particles as the main cause of osteogenic inhibition 7-10. Wear particles directly impair the expression of various collagens, alkaline phosphatase (ALP) activity, osteogenic differentiation and mineralization in osteoblasts 8, 9, 11, 12. Besides, the chemokines and cytokines like IL-1, IL-6, TNF-α, and PGE-2 secreted by macrophages suppress osteoblastic function 10, 13-15. Moreover, osteogenic reduction in titanium-induced osteolysis might be regulated via the Wnt and BMP signaling pathway 16, 17. Therefore, triggering the promotion of osteogenesis could be a promising therapy in the treatment of PPO.

Frankincense is a hard, gelatinous resin exudate found in the trunks of trees belonging to the olive family 18. It is extensively used as a medicinal material in Chinese traditional medicine and Ayurvedic medicine in India 19, 20. The primary chemical constituents of frankincense are terpenoids, volatile oils, arabinose, and xylose, which are collectively known as boswellic acid (BA) compounds due to their carboxyl structures. The main active ingredients in frankincense are α-boswellic acid (α-BA), β-boswellic acid (β-BA), β-BA-derivative acetyl-boswellic acid (ABA), 11-keto-β-boswellic acid (KBA), and acetyl-11-keto-β-boswellic acid (AKBA), among which AKBA is the most active and widely used 21, 22. Several studies have shown that AKBA has anti-inflammatory, anti-tumor, anti-ulcer, anti-asthma, anti-oxidation, analgesic, immunomodulatory, and lipid regulatory effects 23-26. However, relatively few studies have explored the effect of AKBA on bone metabolism. Takada and coworkers found that AKBA enhanced osteoclast apoptosis and suppressed osteoclastogenesis by inhibiting the NF-κB signaling pathway 27. Recently, Bai et al. found an association between AKBA treatment and osteoblast differentiation 28. These findings assumed that AKBA was involved in the regulation of bone tissue; whether AKBA regulated bone formation in titanium-induced osteolysis was unclear.

In this study, we explored the relationship between AKBA treatment and titanium-induced osteogenic reduction. We used titanium particles to induce osteolysis in murine calvaria *in vivo* and incubated them with MC3T3 cells *in vitro*. Our results demonstrated that AKBA treatment promoted bone regeneration following osteolysis induced by titanium particles. The bone regeneration occurred through the AKBA-mediated activation of the GSK-3β/β-catenin signaling pathway and promotion of osteogenic differentiation. Taken together, these results indicate that AKBA is a promising pharmaceutical therapy for the prevention and treatment of PPO.

## Materials and Methods

### Titanium particle preparation

Titanium particles, which were used to stimulate osteolysis, were purchased from Alfa Aesar (#00681, Ward Hill, MA, USA) and processed by Powder-Tech Associates, Inc. (North Andover, MA, USA). Over 95% of the titanium particles had circle diameters smaller than 4 µm. Endotoxins were removed by calcining at 180°C for 12 h and then rinsing in 75% ethanol for 48 h. The concentration of endotoxins in the particles was detected using a Limulus Amoebocyte Lysate assay detection kit (LAL, Biowhittaker, Walkersville, MD, USA). Endotoxin standard (0.1, 0.25, 0.5, 1.0 EU/ml) or 50 μl of the sample (0.1 mg/ml titanium particles after endotoxin removal) were dispensed into the endotoxin-free reaction tubes and incubated in 37°C water bath. Each series of determinations included a blank (50 μl of LAL Reagent Water) plus the four endotoxin standards run in duplicate. Subsequently, 50 μl of LAL and 100 μl of substrate solution (prewarmed 37°C) were added. The reaction was terminated after 10 min by adding 100 μl of stop reagent, and the particles were removed by centrifugation (1200 rpm for 10 min). Subsequently, 200 μl solution from each tube was transferred to a 96-well plate and the absorbance was read at 405 nm. Finally, concentrations of endotoxin were calculated by using a standard curve, and only samples with endotoxin levels <0.02 EU/mL were used 29.

### Animal studies and drug application

A titanium-induced mouse calvaria model was generated as previously described 30, 31. All protocols met the requirements of the National Institutes of Health (NIH) Guide for the Care and Use of Laboratory Animals and the guidelines for animal treatment of the First Affiliated Hospital of Soochow University (approval number: 201709A472). Briefly, 40 eight-week-old male C57BL/6 mice were randomly divided into four groups (n=10/group): (1) sham operation group, treated with physiological saline, (2) model group, treated with 20 mg titanium particles in physiological saline, (3) low AKBA treatment group in which model group mice were treated with 5 mg/kg per day AKBA, and (4) high AKBA treatment group in which model group mice were treated with 20 mg/kg per day AKBA. After anesthetizing and preparing the skin, an incision was made in the middle of the calvaria. In the sham operation group, the surgical incision was stitched closed without additional intervention. In the other groups, 20 mg titanium particles were evenly injected under the periosteum of the biparietal suture. AKBA (Sigma-Aldrich, St. Louis, MO, USA) was dissolved in dimethyl sulfoxide (DMSO) and diluted in physiological saline, and each group received the same amount of DMSO. After 2 days, either physiological saline or AKBA was administered to the model and AKBA groups, respectively, via gastric infusion once a day for two weeks.

### Micro-computed tomography (micro-CT) analysis

The mice were sacrificed after two weeks by cervical dislocation, and the calvaria (n=5/group) were collected and fixed in 4% paraformaldehyde. Surface erosion in the calvaria was measured using Micro-CT (SkyScan1176, Aartselaar, Belgium). The scanning parameter of the isometric resolution was set at 9 μm, and the X-ray energy was set at 80 kV and 100 mA, according to the manufacturer's instructions. Three-dimensional (3D) image reconstructions were taken, and SkyScan software was used for quantitative analysis. Regions of interest (ROI) of equal area (3 mm in diameter) were identified in the center of each calvaria. SkyScan software was used to analyze bone mineral density (BMD), bone volume (BV), bone volume/tissue volume (BV/TV), and the number of pores in the ROI.

### Histological and immunohistochemical analyses

After decalcification in 10% ethylene diamine tetraacetic acid (EDTA, Sigma-Aldrich, St. Louis, MO, USA) for 28 days, the calvaria (n=5/group) were embedded in molten paraffin. The tissues were cut into 6 µm sections and prepared for hematoxylin and eosin (H&E), Masson's trichrome, and tartrate-resistant acid phosphatase (TRAP) staining, following the manufacturer's protocols. Images were captured using an AxioCam HRc microscope (Carl Zeiss, Germany). The bone eroded surface (BES) area ratio and bone thickness (BT) at the closest side of biparietal suture were determined using Image J, and the ratio of osteoclast surface-to- bone perimeter (Oc.S/OB) and the number of osteoclasts to bone surface (N.Oc/BS) were determined using Bioquant Osteo 2017 (BIOQUANT Image Analysis Corporation, Nashville, TN, USA). Blue-colored new bone collagen fiber was observed using Masson's trichrome staining.

Calcein double labeling assay was used to evaluate the dynamic bone formation. Briefly, calcein (Sigma-Aldrich) dissolved in phosphate buffered saline (PBS) was intraperitoneally injected at a concentration of 10 mg/kg on the 1st and 7th days before sacrifice. The calcein double labeling images of undecalcified bone sections were captured using a fluorescence microscope, and mineral apposition rate (MAR) was calculated using Bioquant Osteo 2017.

Expression of alkaline phosphatase (ALP), Osteocalcin (OCN), Osterix, pSer9-GSK-3β, and β-catenin in the calvaria was detected by immunohistochemistry (IHC) using the corresponding primary antibodies (Abcam, ab95462, ab93876, ab22552, ab131097, ab32572). In brief, the sections were dewaxed with xylene and then subjected to gradient hydration and antigen retrieval with hyaluronidase for 1 h at 37°C and pepsin for 25 min at room temperature. The sections were then blocked with second antibody homologous serum for 30 min. Next, the paraffin sections were incubated with primary antibodies at 4°C. After 12 h, the sections were rinsed with PBS and incubated with secondary antibodies for 30 min at room temperature. A DAB Horseradish Peroxidase Color Development Kit (Beyotime, Shanghai, China) was used to induce the chromogenic reaction. IHC-positive cells, which were dark brown and mainly distributed in the periostea and laminae superficialis of the bone lamella, were counted using Bioquant Osteo 2017.

### Cell culture and osteoblast differentiation

The mouse osteoblast cell line MC3T3-E1 was received from the American Type Culture Collection (ATCC, Rockville, MD, USA). After thawing, MC3T3-E1 cells were cultured in Alpha-Minimum Essential Medium (α-MEM) supplemented with 10% FBS and 1% penicillin/streptomycin at 37°C with 5% CO_2_. Osteogenic induction medium consisted of Dulbecco's Modified Eagle Medium (DMEM) supplemented with 10% FBS, 1% penicillin/streptomycin, 100 nM dexamethasone, 10 mM β-glycerophosphate, and 50 mM vitamin C. To simulate the osteolytic microenvironment, 5 μg/cm^2^ titanium particles in medium were added to the cells, and the medium was changed every 3 days. The cells were then treated with low (100 nM) or high (1000 nM) concentrations of AKBA in the presence of titanium particles.

### Cell viability assay

MC3T3-E1 cells were cultured in 96-well plates for 12 h to ensure cell adhesion. To assess the toxicity of AKBA, titanium particles, and ICG-001 on the cells, various concentrations were added and incubated for 1, 3, or 5 days. To evaluate cell viability, Cell Counting Kit-8 (CCK-8; Beyotime) was used, following the manufacturer's protocols. The MC3T3-E1 inhibition was plotted using GraphPad Prism 7.

### Alkaline phosphatase assay

MC3T3-E1 cells were grown in a differentiation-inducing medium for 7 days, and ALP was measured as a phenotypic marker of bone formation. Cells were rinsed 3 times with PBS before fixation in 4% paraformaldehyde at 4°C for 30 min. After washing 3 times with PBS, the cells were incubated in 5-bromo-4-chloro-3-indolyl-phosphate/nitro blue tetrazolium (BCIP/NBT; Beyotime) staining solution for 30 min at room temperature in the dark. ALP-positive cells were counted under a microscope.

### Alizarin red S staining

MC3T3-E1 cells were grown in a differentiation-inducing medium for 14 days, washed 3 times with ddH_2_O, and then fixed in 95% alcohol for 15 min. The cells were then incubated in Alizarin red S (Sciencell, California, USA) solution for 15 min and washed 3 times with ddH_2_O at room temperature before imaging under a microscope. The alizarin red complexed with calcium nodules was dissolved in 5% perchloric acid solution for 30 min at 37°C, and the absorbance was determined at 420 nm.

### RNA extraction and quantitative real-time (qRT)-PCR

MC3T3-E1 cells were grown in a differentiation-inducing medium for 24 hours, and total RNA was isolated using Beyozol reagent (Beyotime). The RNA integrity was determined by separating the RNA on an agarose gel and quality was assessed by measuring the A260/A280 and A260/A230 ratio cutoff higher than 1.8 and 2.0, respectively. Two micrograms of total RNA were used for reverse transcription to synthesize cDNA with dNTP mix and RNase-free H_2_O, following the manufacturer's protocols. qRT-PCR was performed using SYBR1 Premix Ex Taq^TM^ (Takara, Dalian, Japan). The qRT-PCR parameters were as follows: 95.0°C for 30 s, 39 cycles of 95.0°C for 5 s and 60.0°C for 30 s, and 95.0°C for 10 s, followed by a melt curve from 65.0 to 95.0°C at an increment of 0.5°C per 5 s. Three samples were obtained from three independent experiments, and each sample was analyzed in triplicate. The gene expression data were normalized to an internal control (GAPDH) and were analyzed using the comparative 2^-ΔΔCq^ method only when amplification efficiency calculated using the standard curve of the mRNAs of interest and the internal mRNA was 90%~110% 32. GraphPad Prism 7 was used to calculate the data as fold changes compared with the control group. The primer sequences used are shown in Table 1.

### Western blot analysis

MC3T3-E1 cells were grown in a differentiation-inducing medium for 3 days, washed, and then collected with 0.5% trypsin (GIBCO, Grand Island, U.S.). Cells were lysed in radio immunoprecipitation assay (RIPA; Beyotime) buffer for 30 min at 4°C and then the supernatant was collected by centrifugation at 14,800 rpm at 4°C. The supernatant was purified, and total protein concentration quantified using a bicinchoninic acid protein kit (BCA kit; Beyotime). Twenty µg total protein were separated using SDS-PAGE (New Cell & Molecular Biotech Co., Ltd., Suzhou, China) and then transferred to a nitrocellulose filter membrane. After blocking with QuickBlock™ blocking buffer (Beyotime), the membranes were incubated with the appropriate primary antibodies for 12 h at 4°C. Antibodies against Runx2 (1:1000), OCN (1:500), Osterix (1:1000), total GSK-3β (1:2000), pSer9-GSK-3β (1:500), β-catenin (1:5000), and Axin-2 (1:1000) were purchased from Abcam. The membranes were rinsed 3 times with TBS-Tween and then incubated at room temperature with secondary antibody (1:5000) for 1 h. Enhanced chemiluminescence (ECL; Sigma-Aldrich, St. Louis, MO, USA) was used to visualize protein bands, and the relative grey level was analyzed using Image J.

### Immunofluorescence assay

MC3T3-E1 cells were grown on coverslips in a differentiation-inducing medium for 4 h. The cells were then rinsed 3 times with PBS, fixed in 4% paraformaldehyde for 15 min, and permeabilized with 0.2% Triton X-100 for 10 min. Coverslips were blocked with serum for 30 min. Cover glasses were subsequently incubated in primary antibodies against β-catenin (1:250) overnight at 4°C. Coverslips were incubated with FITC-conjugated secondary antibody (1:200, ab6785, Abcam) at room temperature for 2 h in the dark. After washing 3 times with PBS, the coverslips were incubated with DAPI for 1 h and mounted to slides using HelixGen anti-fade fluorescence mounting medium (Beyotime). Coverslips were imaged using a fluorescence microscope.

### Statistics

The data were presented as means ± standard deviation (SD), and SDs reflected differences between three independent *in vitro* experiments. GraphPad Prism 7 was used for statistical analysis. Normal distribution was verified before using parametric tests with D'Agostino & Pearson omnibus normality. Statistical significance was determined by one-way ANOVA with the Tukey's multiple comparisons test. A value of *P*<0.05 was considered statistically significant.

## Results

### AKBA treatment attenuated bone loss and enhanced bone formation in titanium-induced osteolysis

**Figure 1:**
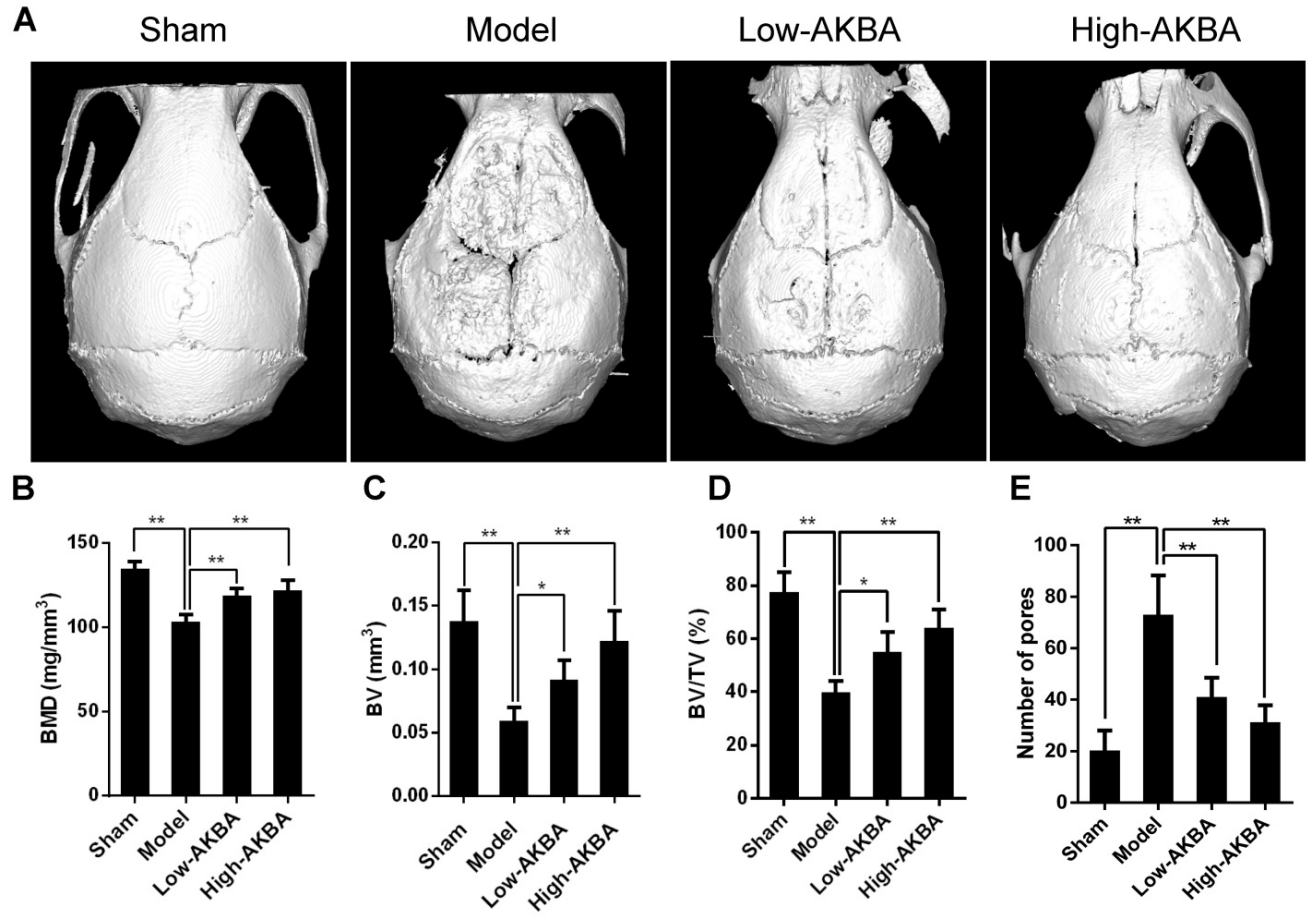
** AKBA treatment attenuated titanium particle-induced bone loss and increased bone mass in a murine calvaria model.** (A) micro-CT reconstruction images, (B) bone mineral density (BMD, mg/mm^3^), (C) bone volume (BV, mm^3^), (D) bone volume/tissue volume (BV/TV, %) and (E) number of pores within the region of interest (ROI). Both low-AKBA (5 mg/kg per day) group and high-AKBA (5 mg/kg per day) group contained 20 mg titanium particles titanium particles. n=5 per group. Data are presented as means ± SD. *P<0.05 and **P<0.01, compared with the model group.

Fig. 1A illustrates that titanium particles enhanced bone loss in murine calvaria, which was markedly attenuated by treatment with AKBA. Moreover, the BMD of the model group was significantly decreased compared with that of the sham group (103.0±4.425 mg/mm^3^ vs. 134.5±4.376 mg/mm^3^, respectively); the decrease of BMDs of the low (118.8±4.293 mg/mm^3^) and high (122.1±5.684 mg/mm^3^) AKBA treatment groups was prevented considerably compared with the model group (Fig. 1B). Also, other parameters, such as the BV, BV/TV, and the number of pores, showed that AKBA eased titanium-induced osteolysis (Fig. 1C-E).

**Figure 2:**
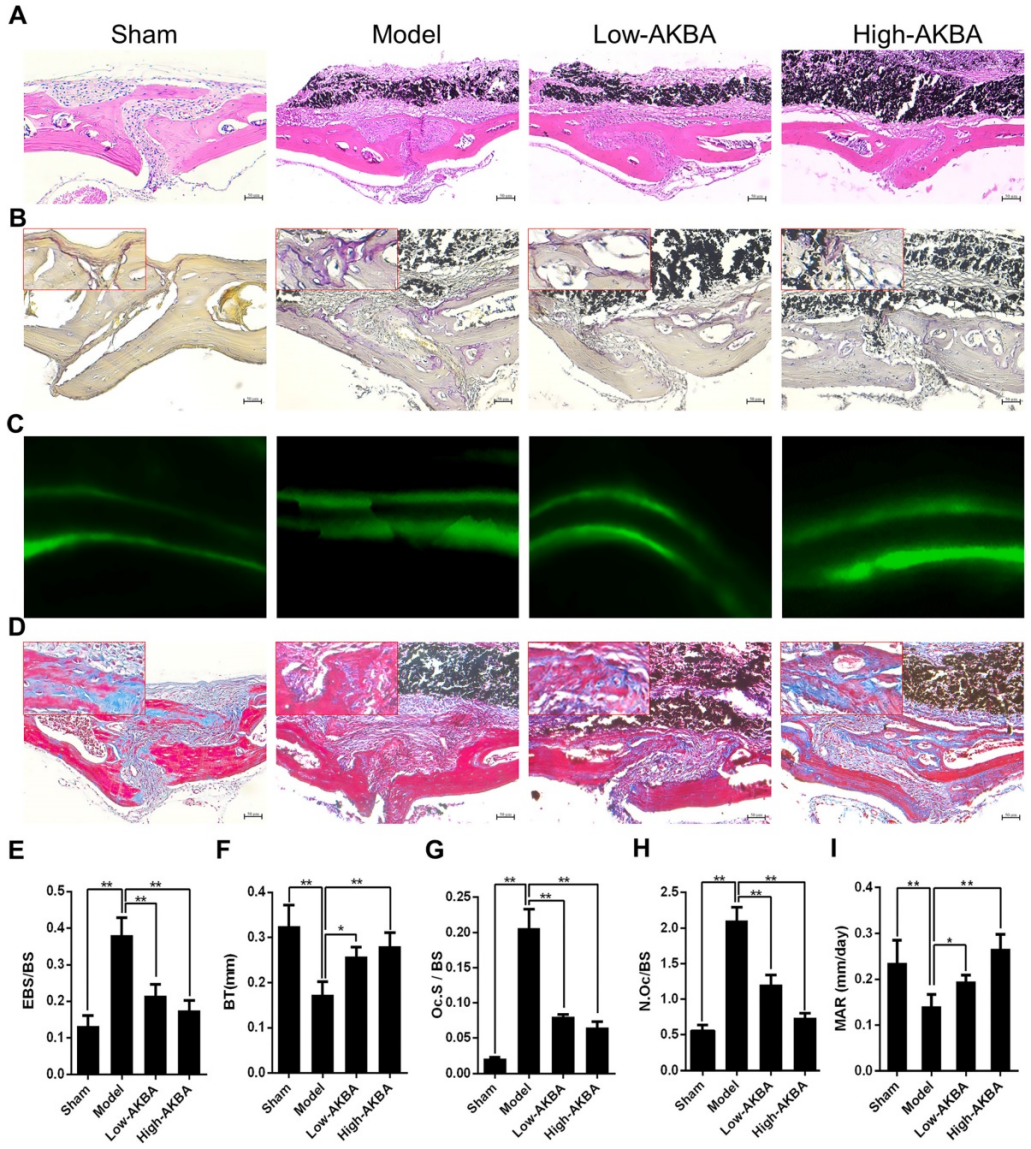
** AKBA treatment attenuated titanium particle-induced osteolysis in histological analysis of calvaria sections.** (A) H&E staining, (B) TRAP staining, (C) Calcein double labeling, (D) Masson's trichrome staining, blue-colored new bone collagen fibers, (E) the ratio of eroded bone surface to bone surface (EBS/BS), (F) bone thickness (BT, mm), (G) osteoclast surface/bone surface (Oc.S/BS), (H) average number of osteoclasts to bone surface (N.Oc/BS), and (I) mineral apposition rate (MAR). Both low-AKBA (5 mg/kg per day) group and high-AKBA (5 mg/kg per day) group contained 20 mg titanium particles titanium particles. n=5 per group. Scale bar=50 μm. Data are presented as means ± SD. *P<0.05 and **P<0.01, compared with the model group.

H&E and TRAP staining were performed to further verify the attenuation of osteolysis upon AKBA treatment (Fig. 2A and B). Bone morphometric analysis of H&E staining indicated that AKBA treatment remarkably reduced the ratio of eroded bone surface to bone surface (EBS/BS; Fig. 2E), as well as prevented the decrease of BT (Fig. 2F) from 0.173±0.029 mm in the model group to 0.258±0.020 mm and 0.281±0.031 mm in low and high AKBA treatment groups, respectively. Likewise, the ratio of osteoclast surface-to-bone surface decreased to 8.06%±0.37% in the low AKBA group and 6.49%±0.93% in the high AKBA group, compared with 20.63%±2.74% in the model group (Fig. 2G). Furthermore, the average number of osteoclasts in the bone surface (N.Oc/BS) was significantly decreased in AKBA-treated mice compared with that in the model group (Fig. 2H).

To observe bone formation in murine calvaria, calcein double labeling and Masson's trichrome staining were performed. The calcein double labeling showed that AKBA treatment significantly enhanced bone formation and increased mineral apposition rate (Fig. 2C&I). Similarly, more new bone collagen fibers were observed in AKBA-treated groups compared to the model group (Fig.2D).

### AKBA treatment eased titanium-induced osteolysis by enhancing osteogenesis

**Figure 3:**
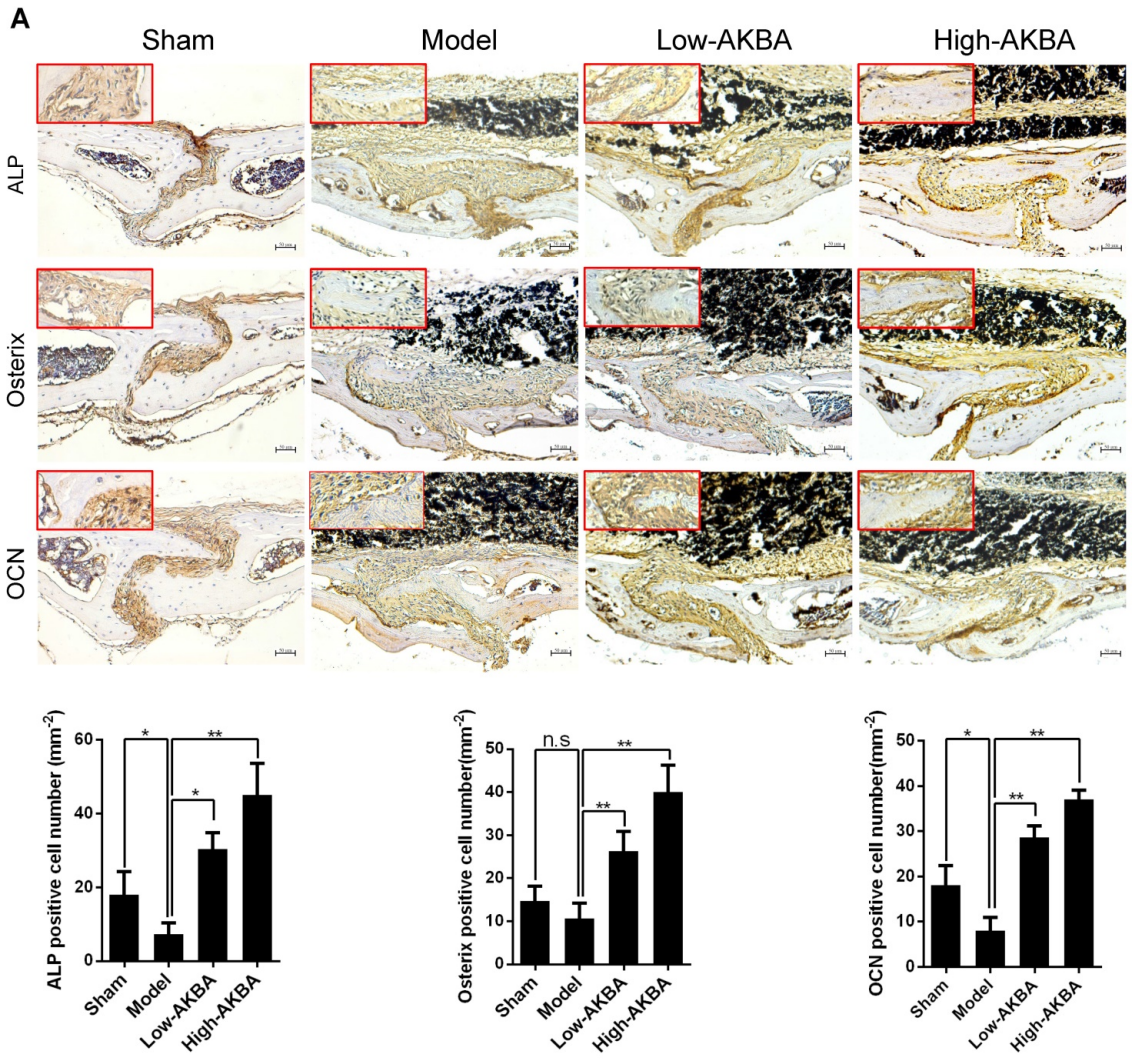
** AKBA treatment enhanced new bone regeneration in titanium particle-stimulated osteolysis.** (A-C) IHC for ALP, Osterix, and OCN. (D-F) Quantification of the number of positive cells. Both low-AKBA (5 mg/kg per day) group and high-AKBA (5 mg/kg per day) group contained 20 mg titanium particles titanium particles. n=5 per group. Scale bar=50 μm. Data are presented as means ± SD, *P<0.05 and **P<0.01; n.s. indicates no statistical significance.

To further verify whether AKBA treatment enhanced osteogenesis in titanium-induced osteolysis, IHC staining was used to measure the expression of osteogenic markers. AKBA treatment significantly enhanced osteogenesis in titanium-induced osteolysis, as evidenced by increased expression of the osteogenic markers ALP, Osterix, and OCN (Fig. 3A-C). However, fewer IHC-positive cells were observed in the model group compared with either the sham group or AKBA treatment groups. These results suggested that titanium particles inhibited the expression of osteogenic markers, while AKBA treatment increased their expression (Fig. 3D-F).

### AKBA treatment alleviated titanium-induced osteogenic reduction *in vitro*

**Figure 4:**
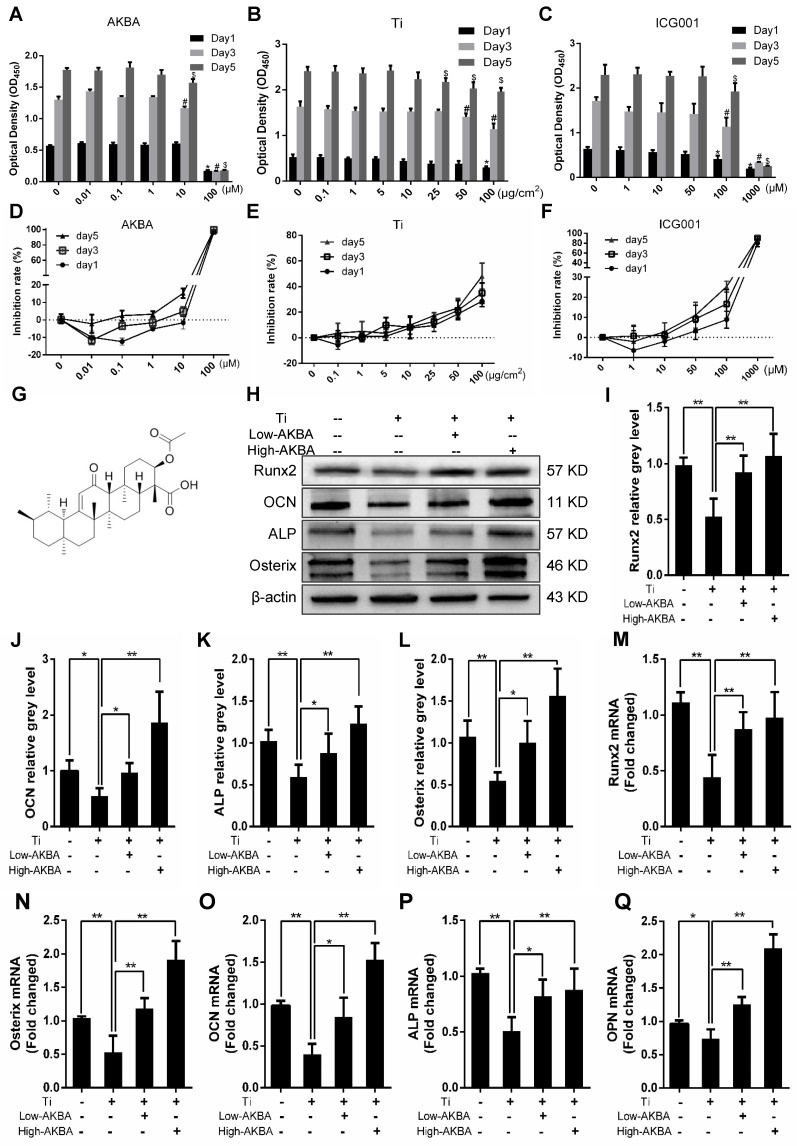
** AKBA treatment attenuated titanium particle-induced inhibition of osteogenic differentiation in MC3T3-E1 cells.** (A-C) Cell viability after 1, 3, or 5 days of treatment. Viability was determined using a CCK-8 assay. *P<0.05, compared with 0 μM treatment (day 1), ^#^*P*<0.05, compared with 0 μM treatment (day 3), and ^$^*P*<0.05, compared with 0 μM treatment (day 5). (D-F) Inhibition rate of MC3T3-E1 cells. (G) Chemical structure of AKBA. (H-L) Runx2, OCN, Osterix, and ALP protein expression levels. (M-Q) Runx2, Osterix, OCN, ALP, and OPN mRNA expression levels. Cell differentiation was induced for 1 and 3 days in qRT-PCR and Western blotting, respectively. Relative grey levels were analyzed using Image J. Both low-AKBA (100 nM) group and high-AKBA (1000 nM) group contained 5 μg/cm^2^ titanium particles. Data are presented as means ± SD. n=6: six samples were obtained from six wells in a single experiment for the CCK-8 assay. n=6: three samples were obtained from three independent experiments, and each sample was collected from three wells in a single experiment and analyzed in duplicates by Western blotting. n=9: three samples were obtained from three independent experiments, and each sample was collected from three wells in a single experiment and analyzed in triplicate by qRT-PCR. *P<0.05 and **P<0.01, compared with the titanium particle group.

The CCK-8 cell viability assay was used to determine whether AKBA, titanium particles, and ICG-001 had any cytotoxic effects on MC3T3-E1 cells. The results indicated that cell viability was unaffected by treatment with concentrations below 10 μM of AKBA (Fig. 4A and D), 10 μg/cm^2^ of titanium particles (Fig. 4B and E) and 50 μM of ICG-001 (Fig. 4C and F) over 1, 3, and 5 days.

**Figure 5:**
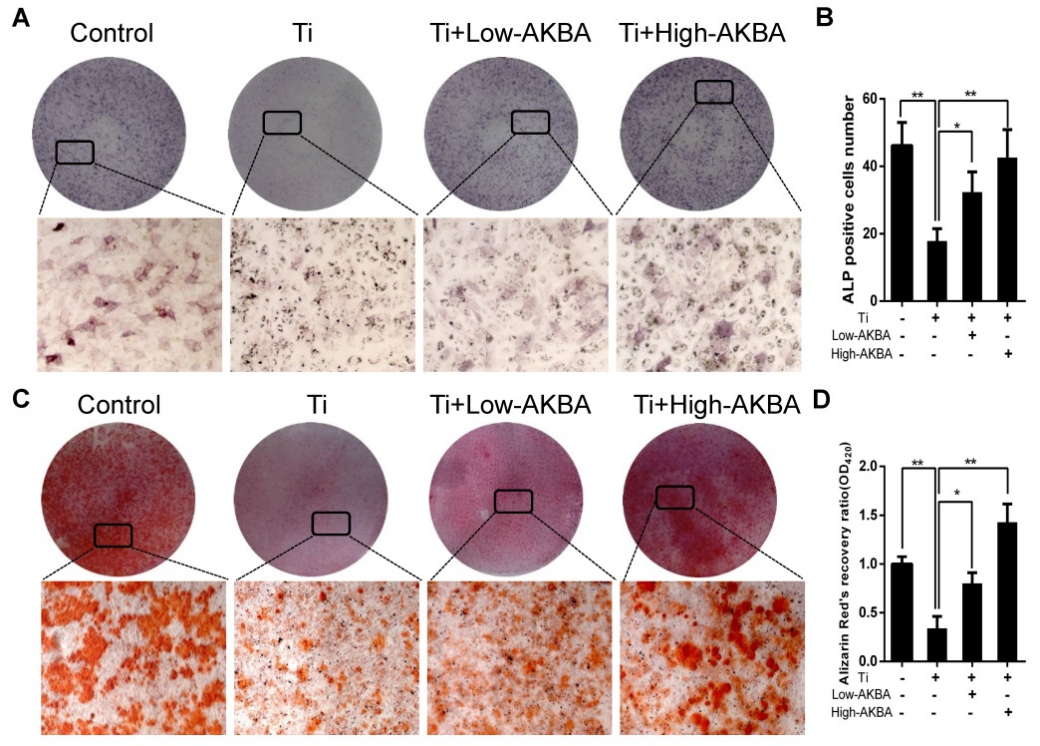
AKBA treatment alleviated titanium particle-induced inhibition of osteogenic differentiation and mineralization in MC3T3-E1 cells. (A) ALP staining at day 7 after osteogenic differentiation. (B) The number of ALP-positive cells in MC3T3-E1 cells. (C) Alizarin Red S (ARS) staining of MC3T3-E1 cells at day 14 after osteogenic differentiation. (D) Semi-quantitative analysis of ARS staining. Both low-AKBA (100 nM) group and high-AKBA (1000 nM) group contained 5 μg/cm^2^ titanium particles. Data are presented as means ± SD. n = 9: nine samples were obtained from three independent experiments, and each sample was collected from one well in a single experiment for the ALP staining and ARS staining. n= 9: three samples were obtained from three independent experiments, and each sample was collected from three wells in a single experiment and analyzed in triplicate for semi-quantitative analysis of ARS. *P<0.05 and **P<0.01, compared with the titanium particle-treated group.

Differentiated MC3T3-E1 cells were stimulated by 5 μg/cm^2^ titanium particles with or without low (100 nM) or high (1000 nM) concentrations of AKBA for 3 days. Western blot analysis showed that the expression of ALP, Osterix, OCN, and Runx2 was increased in AKBA-treated cells (Fig. 4H-L), indicating that treatment mitigated titanium-induced osteogenic reduction by enhancing the expression of osteogenic proteins. Also, the qRT-PCR analysis demonstrated that AKBA treatment resulted in a significant increase in the mRNA levels of runt-related transcription factor 2 (Runx2), Osterix, OCN, Osteopontin (OPN), and ALP (Fig. 4M-Q). ALP staining revealed that titanium particles inhibited the osteogenic differentiation of MC3T3-E1 cells which was alleviated by AKBA treatment in a dose-dependent manner (Fig. 5A and B). Consistent with this, Alizarin red S staining (ARS) demonstrated that 100 nM and 1000 nM AKBA treatment profoundly stimulated cell mineralization by approximately 224.3% and 324.3%, respectively, compared to cells treated with titanium particles only (Fig. 5C and D).

### AKBA treatment alleviated titanium-induced osteogenic reduction in osteolysis by activating the GSK-3β/β-catenin signaling pathway *in vitro* and *in vivo*

**Figure 6:**
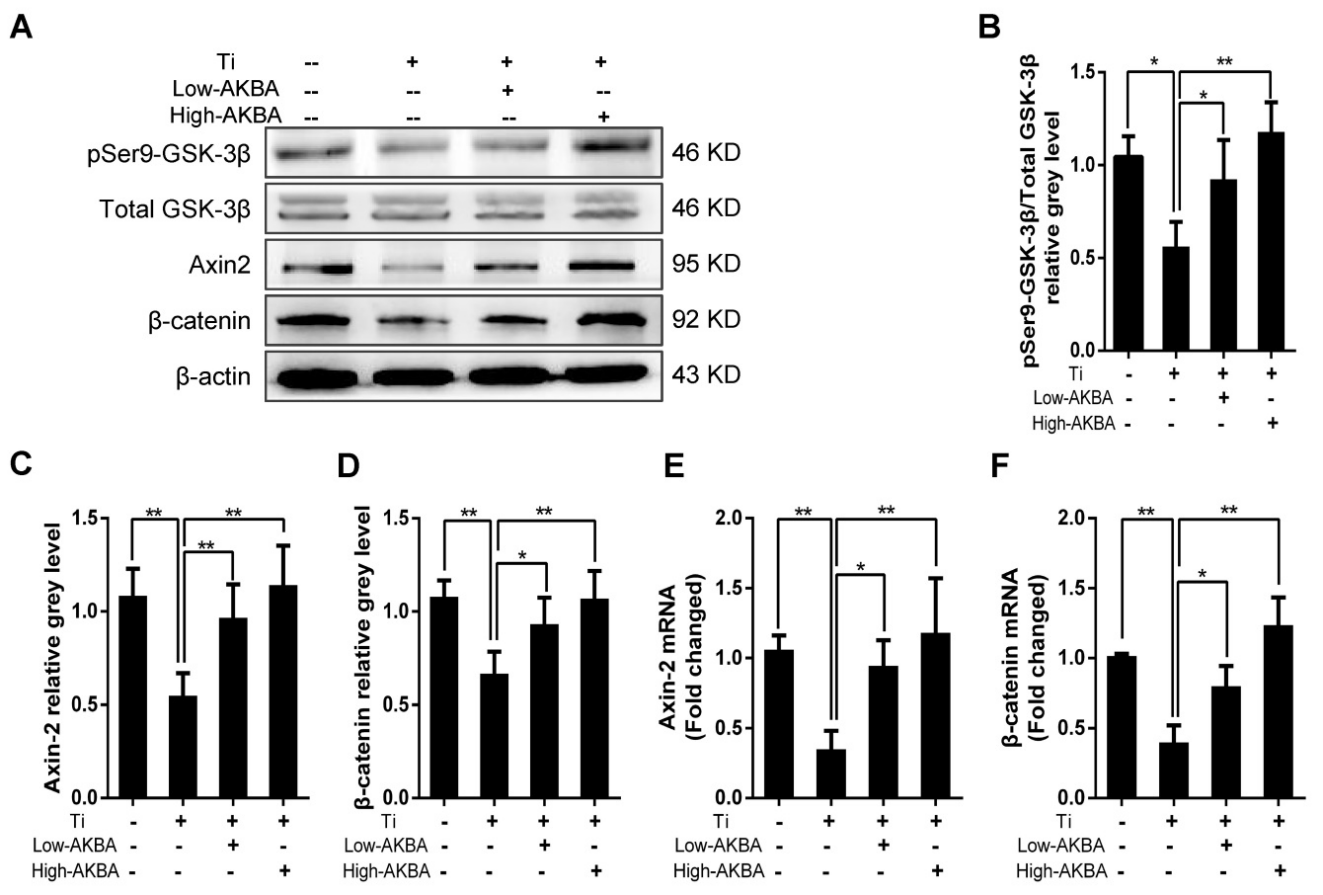
** AKBA treatment rescued the titanium particle-induced inhibition of β-catenin in MC3T3-E1 cells.** (A) Western blot analysis of expression levels of pSer9-GSK-3β, β-catenin, and Axin-2. (B-D) The relative grey levels of pSer9-GSK-3β/total-GSK-3β, β-catenin, and Axin-2. (E, F) qRT-PCR analysis of the mRNA expression levels of pSer9-GSK-3β, β-catenin, and Axin-2. Both low-AKBA (100 nM) group and high-AKBA (1000 nM) group contained 5 μg/cm^2^ titanium particles. Data are presented as means ± SD. n=6 or 9 for Western blotting and qRT-PCR, respectively. *P<0.05 and **P<0.01, compared with the titanium particle-treated group.

To investigate the potential molecular mechanisms underlying the osteogenic effects of AKBA treatment, we examined the expression of key markers involved in relevant signaling pathways and MC3T3-E1 differentiation. As expected, Western blot and qRT-PCR analyses showed that the expression of β-catenin and Axin-2 was inhibited by the titanium particles. However, β-catenin and Axin-2 levels were markedly increased in cells treated with AKBA compared to cells treated with titanium particles only. Western blot analysis of pSer9-GSK-3β and GSK-3β showed that AKBA treatment decreased the ratio of pSer9-GSK-3β to GSK-3β induced by titanium particles. These results indicated that the GSK-3β/β-catenin signaling pathway may have mediated the protective effects of AKBA treatment (Fig. 6).

**Figure 7:**
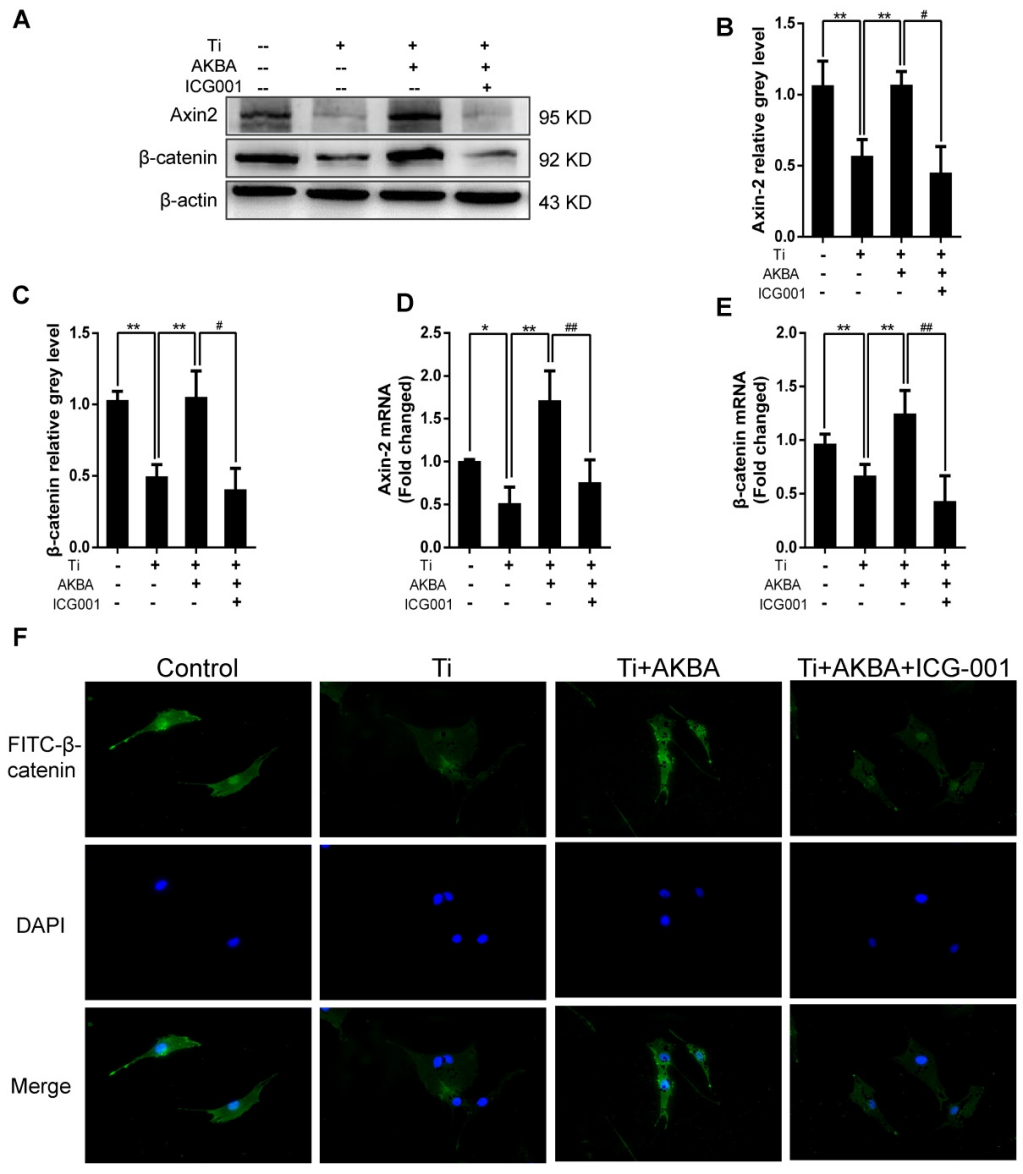
** ICG-001 treatment reversed the effects of AKBA on the activity of β-catenin in MC3T3-E1 cells.** (A-C) Western blot and (D, E) qRT-PCR analysis of the protein and mRNA levels of β-catenin and Axin-2. (F) Cellular immunofluorescence of β-catenin in MC3T3-E1 cells. Cell differentiation was induced for 24 h. AKBA group contained 5 μg/cm^2^ titanium particles and 1000 nM AKBA, and ICG-001 group contained 5 μg/cm^2^ titanium particles, 1000 nM AKBA and 20 μM ICG-001. Data are presented as means ± SD. n=6 or 9 for western blotting and qRT-PCR, respectively. n=9: nine samples were obtained from three independent experiments, and each sample was collected from one well in a single experiment for immunofluorescence assay. *P<0.05 and **P<0.01, compared with the model group. ^#^*P*<0.05,**^##^***P*<0.01, compared with the AKBA treatment group.

Indocyanine green-001 (ICG-001, Selleck), a specific inhibitor of the Wnt/β-catenin signaling pathway, was used to verify the role of the GSK-3β/β-catenin pathway. We first confirmed that ICG-001 could inhibit osteoblastic differentiation and mineralization via inhibiting Wnt/β-catenin signaling pathway as shown in Fig. S1 and Fig. S2. Strikingly, treatment with 20 µM ICG-001 (contained 5 μg/cm^2^ titanium particles and 1000 nM AKBA) abolished the AKBA-mediated rescue of the titanium particle-induced inhibition of the Wnt/β-catenin signaling pathway. Western blot (Fig. 7A-C) and qRT-PCR (Fig. 7D and E) analyses indicated that ICG-001 inhibited the expression of β-catenin and Axin-2. Cellular immunofluorescence assays also indicated that AKBA treatment promoted the activation of β-catenin and increased its nuclear translocation. However, these effects were suppressed in the presence of ICG-001 (Fig. 7F).

**Figure 8:**
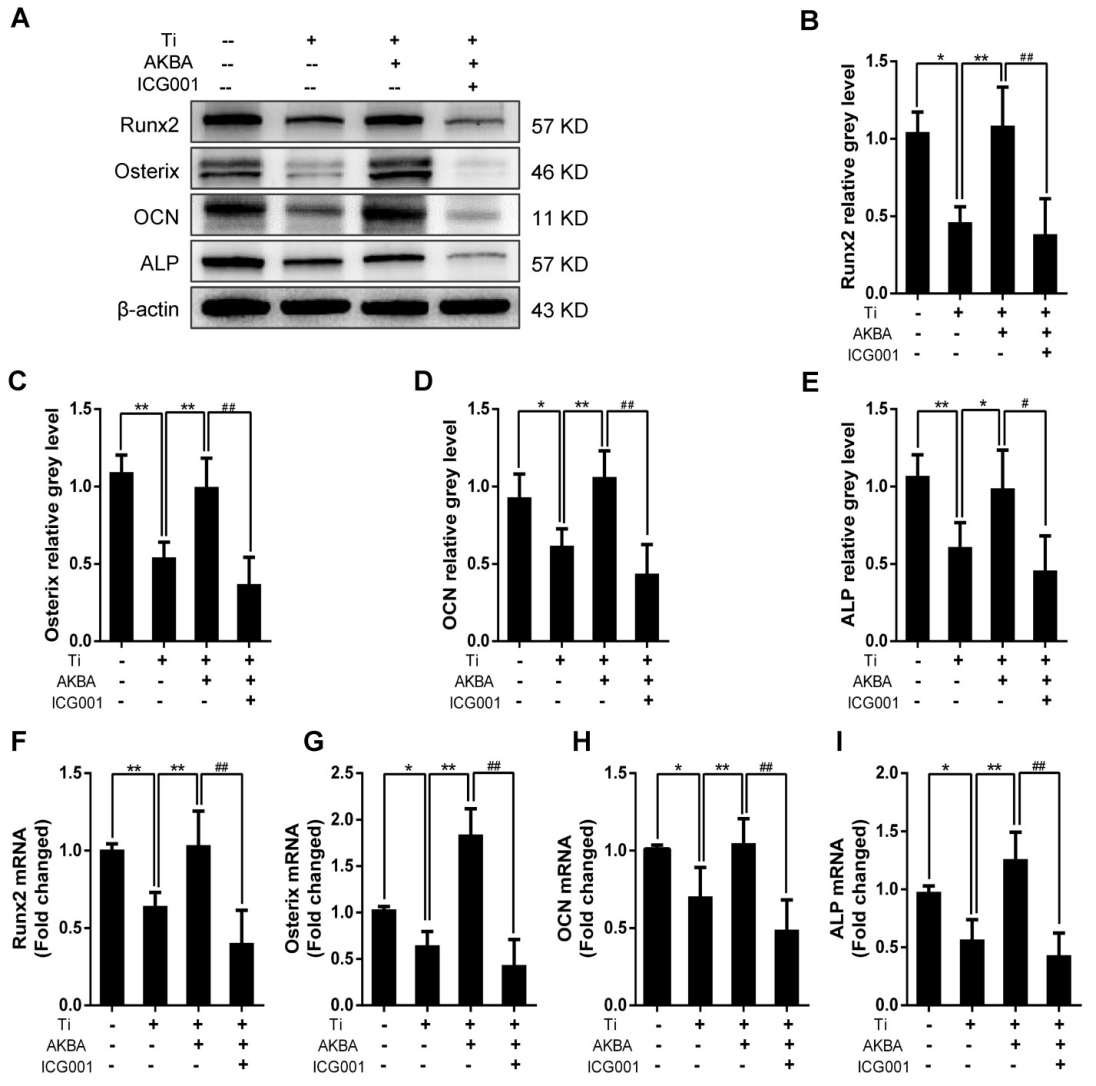
** ICG-001 treatment reversed the effects of AKBA on osteogenic differentiation in MC3T3-E1.** (A-E) Western blot analysis of expression levels of Runx2, Osterix, OCN, and ALP. (F-I) qRT-PCR analysis of the mRNA expression levels of Runx2, Osterix, OCN, and ALP. AKBA group contained 5 μg/cm^2^ titanium particles and 1000 nM AKBA, and ICG-001 group contained 5 μg/cm^2^ titanium particles, 1000 nM AKBA and 20 μM ICG-001. Data are presented as means ± SD. n=6 or 9 for western blot and qRT-PCR, respectively. *P<0.05 and **P<0.01, compared with the model group.**^ #^***P*<0.05 and ^##^*P*<0.01, compared with the AKBA treatment group.

**Figure 9:**
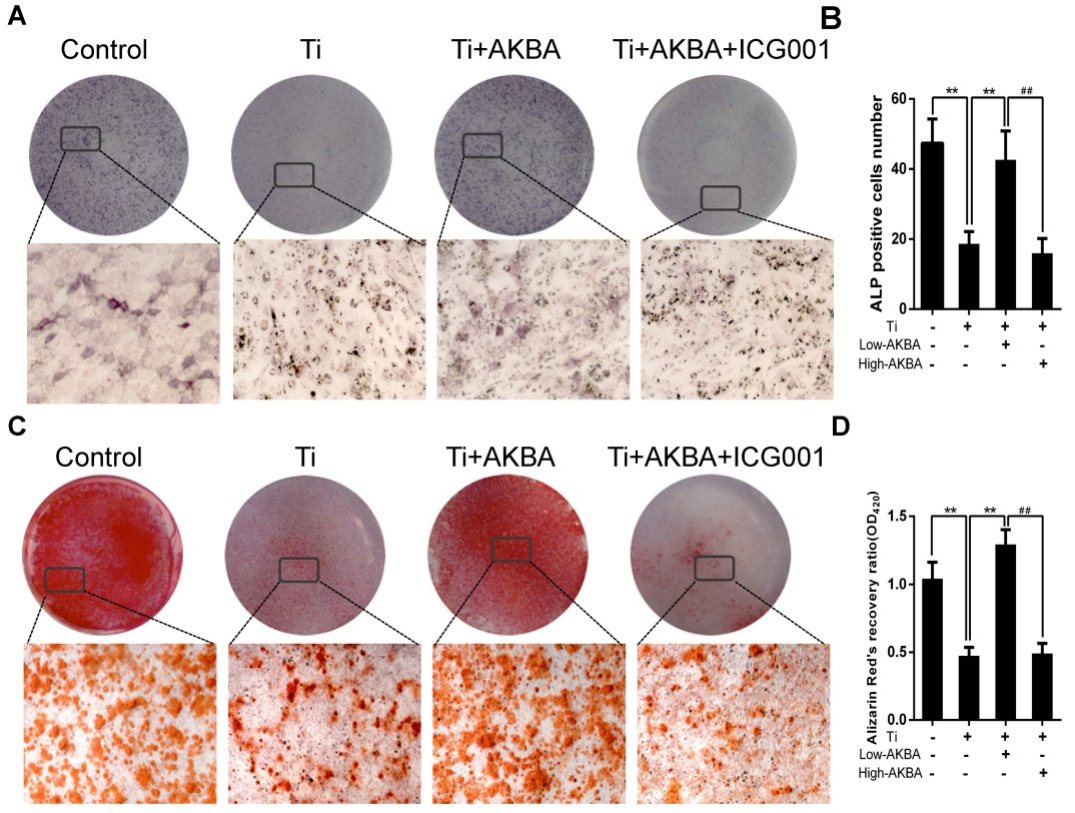
** ICG-001 treatment reversed the effects of AKBA on osteogenic differentiation and mineralization in MC3T3-E1.** (A) ALP staining in MC3T3-E1 cells. (B) The number of ALP-positive cells. (C) ARS staining in MC3T3-E1 cells. (D) Semi-quantitative analysis of ARS staining. AKBA group contained 5 μg/cm^2^ titanium particles and 1000 nM AKBA, and ICG-001 group contained 5 μg/cm^2^ titanium particles, 1000 nM AKBA and 20 μM ICG-001. Data are presented as means ± SD, n = 9 for ALP staining, ARS staining and semi-quantitative analysis of ARS. *P<0.05 and **P<0.01, compared with the model group. **^#^***P*<0.05 and **^##^***P*<0.01, compared with the AKBA treatment group.

Western blotting (Fig. 8A-E) and qRT-PCR (Fig. 8F-I) showed drastic decrease in the expression of osteogenic markers by ICG-001 treatment. As expected, ALP staining and the number of ALP-positive cells (Fig. 9A and B) were consistent with the number of calcium nodules detected by ARS staining, which were markedly reduced in cells treated with ICG-001 compared to cells treated with AKBA alone (Fig. 9C and D). Overall, ICG-001 severely inhibited the protective effect of AKBA on β-catenin activity. These findings indicated that AKBA treatment alleviated the inhibition of the GSK-3β/β-catenin signaling pathway caused by titanium particles, while ICG-001 treatment inhibited the protective effect of AKBA in titanium-induced osteogenic inhibition.

**Figure 10:**
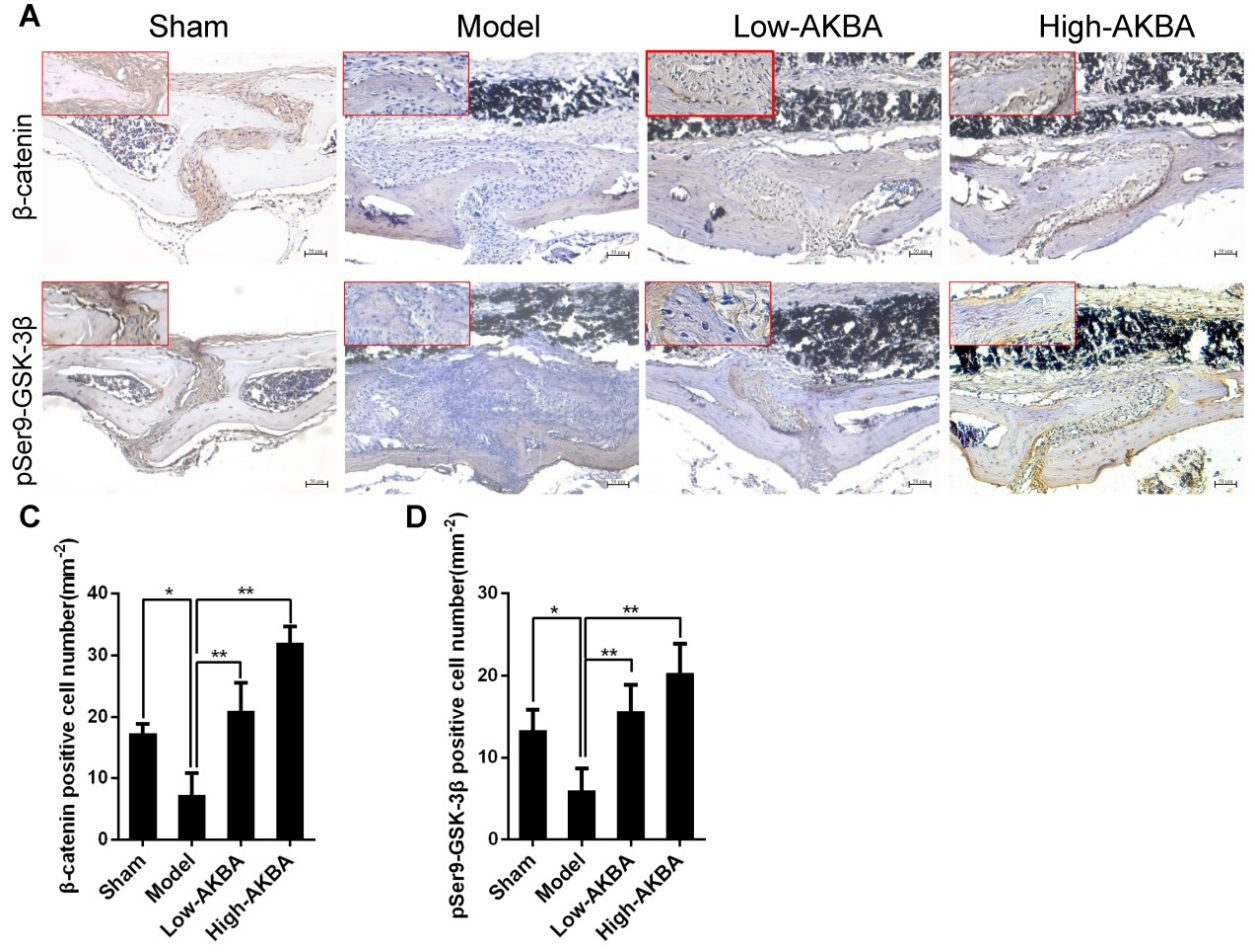
** AKBA treatment activated the GSK-3β/β-catenin signaling pathway *in vivo*.** (A, B) IHC analysis of the expression of pSer9-GSK-3β and β-catenin. (C, D) Quantification of the number of positive cells. Both low-AKBA (5 mg/kg per day) group and high-AKBA (5 mg/kg per day) group contained 20 mg titanium particles titanium particles. n=5 per group. Scale bar=50 μm. Data are presented as means ± SD. *P<0.05 and **P<0.01.

To further confirm that AKBA treatment improved titanium particle-induced osteogenic reduction via the GSK-3β/β-catenin signaling pathway in murine calvaria, IHC staining of pSer9-GSK-3β and β-catenin was performed. Intense pSer9-GSK-3β and β-catenin staining was observed in AKBA-treated mice, compared with the low expression observed in the model mice (Fig. 10A and B). The semi-quantitative analysis showed that titanium particle-stimulation reduced the expression of β-catenin and pSer9-GSK-3β compared with that in the control group. Treatment with AKBA reversed the effects of titanium particle stimulation (Fig. 10C and D). These data indicated that AKBA mitigated titanium-induced osteogenic reduction in osteolysis via activation of the GSK-3β/β-catenin signaling pathway *in vivo*.

## Discussion

Wear particles play an important biological role in the occurrence of PPO, an unavoidable problem that results in implant failure and revision surgery 33-35. Proinflammatory and osteoclastogenic cytokines secreted by macrophages, fibroblasts, lymphocytes, and osteoclasts may break the balance between bone formation and bone resorption, resulting in PPO 36. Previous studies have focused on anti-inflammatory, osteoclastogenesis, and biomaterial improvement 37-39. However, inhibiting inflammation caused by wear particles around the prosthesis has proven to be insufficient for the treatment of osteolysis 40. Moreover, the direct exposure of osteoblasts to wear particles is another significant cause of PPO 16, 17, 41, 42. Some studies have found that the expression levels of inflammatory and bone resorption factors were up-regulated and of bone formation-related factors down-regulated in osteoblasts exposed to wear particles, resulting in a decrease in osteogenesis 13, 14, 43. Alleviating the wear particle-induced reduction of periprosthetic osteogenesis is a key factor in the prevention and treatment of PPO.

BAs are pharmacologically active compounds of the pentacyclic triterpenes of different boswellia species. Thus far, over 12 kinds of BAs have been identified, though only KBA and AKBA have significant pharmacological activity 21. AKBA, which is more active than KBA, has been found to inhibit host inflammatory response, mitigating inflammation-induced damage to cartilage, bronchial, and intestinal tissues. Furthermore, AKBA has known anti-tumor, anti-ulcer, anti-asthma, anti-oxidation, immunomodulatory, and lipid regulatory effects 18, 25. AKBA treatment abolished osteoclastogenesis and promoted osteoblastic differentiation through the regulation of NF-κB activity 27, 28. In the current study, we observed that AKBA reversed titanium particle-induced inhibition of osteoblast formation. Also, we showed, for the first time, that AKBA mitigated titanium particle-induced osteolysis and stimulated osteogenesis.

Previous studies have reported that the Wnt/β-catenin signaling pathway plays a vital role in bone formation, and the activation of β-catenin has been shown to promote osteoblast proliferation, differentiation, and maturation 44-46. The Wnt/β-catenin signaling pathway has been shown to be key to the homeostatic maintenance of bone tissue and, as such, has become a promising target of osteoporosis and rheumatoid arthritis therapies 47, 48. GSK-3β, a serine/threonine protein kinase, plays a significant part in cell growth, differentiation, apoptosis, stem cell maintenance, and tumor formation 16, 49. Also, GSK-3β can phosphorylate β-catenin, causing its inactivation and degradation in the cytoplasm. Inhibition of GSK-3β by phosphorylation of Ser9 can activate the classical Wnt/β-catenin signaling pathway, further improving osteogenesis 16, 50, 51. Our study showed that AKBA treatment resulted in marked activation of the GSK-3β/β-catenin signaling pathway and mitigated titanium particle-induced osteogenic inhibition. Western blot, qRT-PCR, and IHC analyses further demonstrated that AKBA treatment significantly up-regulated the expression of β-catenin, Axin-2, and inactivated GSK-3β through phosphorylation at Ser 9.

ICG-001, which specifically binds the cAMP-response element binding protein (CREB)-binding protein (CBP) to disrupt its interaction with β-catenin and inhibit CBP function as a coactivator of Wnt/β-catenin-mediated transcription 52, 53, impaired the osteogenic function of MC3T3-E1 cells. Immunofluorescence analysis indicated that AKBA treatment promoted nuclear translocation of β-catenin following titanium particle-induced cytoplasmic localization. However, ICG-001 treatment reversed the effects of AKBA on the activity of β-catenin in MC3T3-E1 cells. Collectively, these results demonstrated that AKBA treatment alleviated the titanium-induced inhibition of bone regeneration via activation of the GSK-3β/β-catenin signaling pathway.

In our previous study, we reported that titanium particles inhibited osteoblastic function and induced bone destruction by regulating GSK-3β that could be alleviated by lithium chloride, a selective inhibitor of GSK-3β. However, GSK-3β, a serine/threonine protein kinase, plays a significant role in numerous vital biological processes, and deletion of GSK-3β gene impaired synaptic plasticity and memory, leading to hypertrophic cardiomyopathy and even death during embryonic development 54-56. Besides, the long-term clinical application of lithium chloride as an inhibitor of GSK-3β is accompanied by acute toxic effects 57, 58. In our current study, we observed that AKBA eased osteolysis by enhancing bone formation, which may be associated with the regulation of GSK-3β. However, the involvement of other osteogenic signaling pathways cannot be ruled out and should be investigated in the future. Additionally, inflammation and osteoclastic resorption are two key factors involved in PPO. Some studies have shown that AKBA has anti-inflammatory effect and regulation of osteogenic and osteoclastogenic functions 27, 28, 59. Thus, AKBA may be a promising drug to prevent and treat PPO by enhancing osteogenesis and anti-inflammatory impact as well as inhibiting osteoclastogenesis.

In the future, we plan to further explore various molecular aspects of AKBA's positive impact on osteogenesis. For example, TRAP staining showed that AKBA treatment could reduce the number of osteoclasts surrounding osteolytic areas. Besides, osteoblasts could enhance the expression and secretion of OPG via Wnt/β-catenin signaling pathway, and subsequently suppress osteoclastogenesis via OPG/RANKL/RANK pathway 60. It would, therefore, be essential to get a better understanding of the direct and/or indirect regulation of osteoclasts by AKBA to attenuate titanium particle-induced osteolysis. Also, inflammation plays a key role in titanium-induced osteolysis, and inflammatory cytokines could not only enhance osteolysis directly, but could inhibit osteogenesis by suppressing the expression of collagen I and osteocalcin 13, 61, 62. AKBA has been shown to exert anti-inflammatory effects on other tissues including bone and cartilage tissues 23, 63. Thus, it is important to clarify the potential anti-inflammatory role of AKBA in mitigating titanium-induced osteolysis. Finally, in our current study, we observed that AKBA eased osteolysis by enhancing bone formation, which may be associated with the regulation of GSK-3β/β-catenin signaling pathway. However, the involvement of other osteogenic signaling pathways cannot be ruled out and should be investigated in the future.

## Conclusion

Our study explored the effect of AKBA treatment on osteogenic reduction in titanium-induced osteolysis. AKBA treatment was found to attenuate titanium-induced osteogenic inhibition which was regulated through the activation of the GSK-3β/β-catenin signaling pathway (Graphical Abstract). These results provide a deeper insight into whether and how AKBA regulates bone metabolism and demonstrate that AKBA is a potential new target for the treatment of wear particle-induced PPO.

## Supplementary Material

Supplementary figures and tables.

## Figures and Tables

**Table 1:** Primer sequence

	Primer sequence		
mRNA	Forward (5'-3')	Reverse (5'-3')	
GAPDH	TGACCTCAACTACATGGTCTACA	CTTCCCATTCTCGGCCTTG	
OCN	AAGCAGGAGGGCAAAAGGT	TTTGTAGGCGGTCTTCAAGC	
Runx2	AGAGTCAGATTACAGATCCCAGG	TGGCTCTTCTTACTGAGAGAGG	
Osterix	ACCCCAAGATGTCTATAAGCCC	CGCTCTAG CTCCTGACAGTTG	
ALP	GGCTGGAGATGGACAAA TTCC	CCGAGTGGTAGTCACAATGCC	
OPN	CACT CCAATCGTCCCTACAGT	CTGGAAACTCCTAGACTTTGACC	
β-catenin	GGAAAGGAGGCACAAAGAAGC	CCCCTTAGGCACTAGGAGC	
Axin-2	ACCAGGATGGTGCATACCTCT	CCCATTACAAGCAAACCAGAAGT	
			

GADPH: glyceraldehyde-3-phosphate dehydrogenase; OCN: osteocalcin; Runx2: runt-related transcription factor 2; ALP: alkaline phosphatase; OPN: osteopontin.

## Abbreviations

ABA: β-BA-derivative acetyl-boswellic acid
AKBA: acetyl-11-keto-β-boswellic acid
ALP: alkaline phosphatase
α-BA: Alpha-boswellic acid
α-MEM: Alpha-Minimum Essential Medium
BA: boswellic acid
BCIP/NBT: 5-bromo-4-chloro-3-indolyl-phosphate/nitro blue tetrazolium
BES: bone eroded surface
BMD: bone mineral density
BT: bone thickness
BV: bone volume
BV/TV: bone volume/tissue volume
β-BA: beta-boswellic acid
CREB: cAMP-response element binding protein
DMEM: Dulbecco's Modified Eagle Medium
DMSO: dimethyl sulfoxide
ECL: enhanced chemiluminescence
EDTA: ethylene diamine tetraacetic acid
GADPH: glyceraldehyde-3-phosphate dehydrogenase
GSK-3β: glycogen synthase kinase-3β
H&E: hematoxylin and eosin
ICG-001: indocyanine green-001
IHC: immunohistochemistry
IL-1β: interleukin 1β
KBA: 11-keto-β-boswellic acid
MAR: mineral apposition rate
NF-κB: nuclear factor-κB
N.Oc/BS: the average number of osteoclasts to bone surface
OCN: osteocalcin
Oc.S/OB: the ratio of osteoclast surface-to-bone surface
OPN: osteopontin
PBS: phosphate buffered saline
PPO: peri-prosthetic osteolysis
qRT-PCR: quantitative real-time polymerase chain reaction
RANKL/RANK/OPG: receptor activator for nuclear factor-κB ligand/ receptor activator for nuclear factor-κB/ osteoprotegerin
RIPA: radio immunoprecipitation assay
Runx2: runt-related transcription factor 2
TJA: total joint arthroplasty
TNF-α: tumor necrosis factor-α
TRAP: tartrate-resistant acid phosphatase
