# Supplementary material for: Acetyl-11-keto-β-boswellic acid attenuates titanium particle-induced osteogenic inhibition via activation of the GSK-3β/β-catenin signaling pathway
Source: Theranostics. 2019 Sep 23;9(24):7140–55. doi: 10.7150/thno.35988 (PMC6831297; doi:10.7150/thno.35988)
Supplement: Supplementary file 1 — Supplementary figures and tables. [file thnov09p7140s1.pdf]

Figure. S1

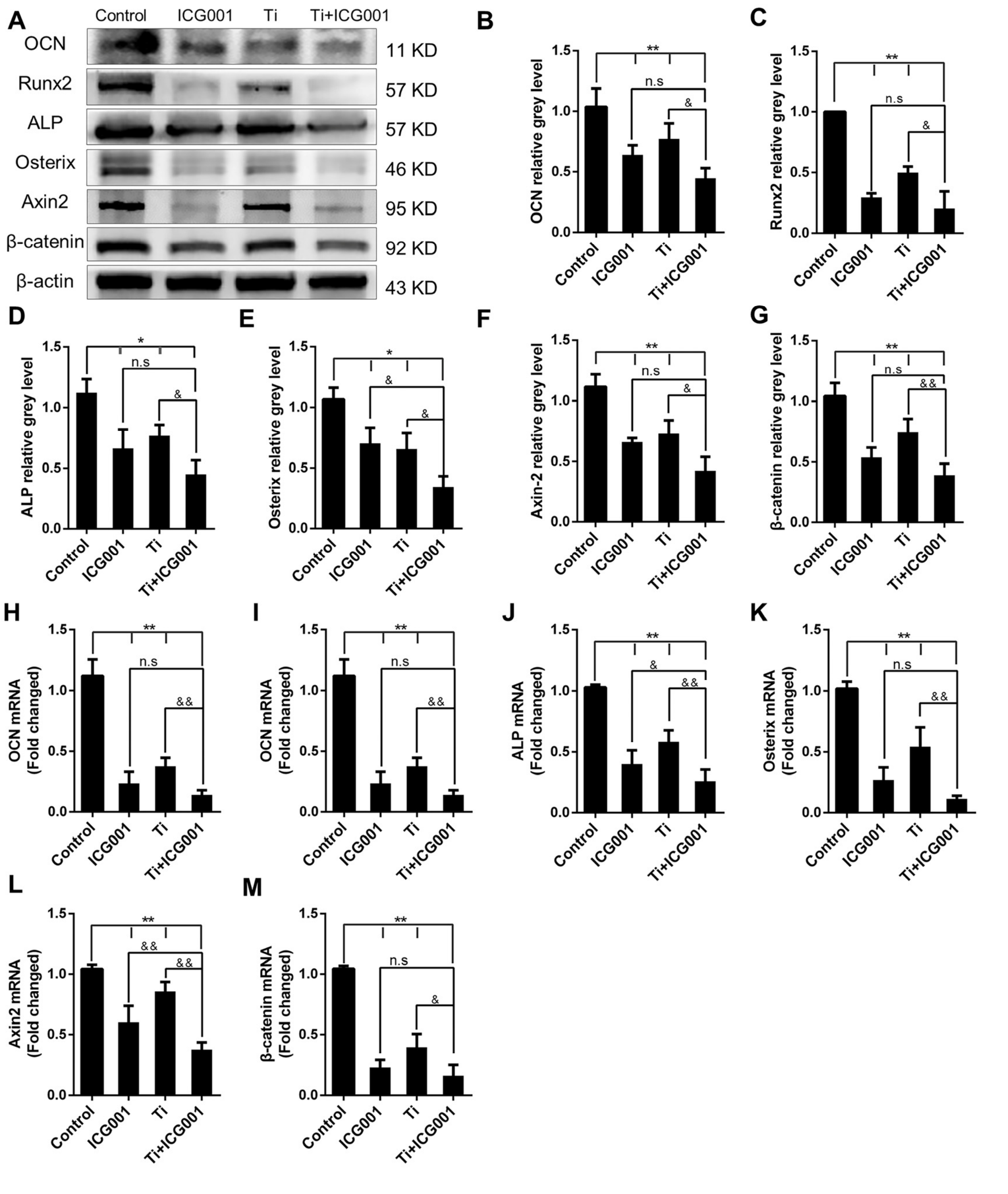

Figure. S1. ICG-001 blocked osteogenic function via inhibiting Wnt/β-catenin signaling pathway whether Ti was added or not. (A) Western blot analysis of expression levels of OCN, Runx2, ALP, Osterix, Axin-2 and β-catenin. (B-G) The relative grey levels. (H-M) qRT-PCR analysis of the mRNA expression levels of OCN, Runx2, ALP, Osterix, Axin-2 and β-catenin. The concentration of titanium particles and ICG-001 was 5 μg/cm^2 and 20 μM, respectively. Data are presented as means ± SD. n=6 or 9 in western blot and in qRT-PCR, respectively. *P<0.05 and **P<0.01,

compared with the control group. &*P*<0.05 and &&*P*<0.01, compared with the Ti+ICG-001 group.

Figure. S2

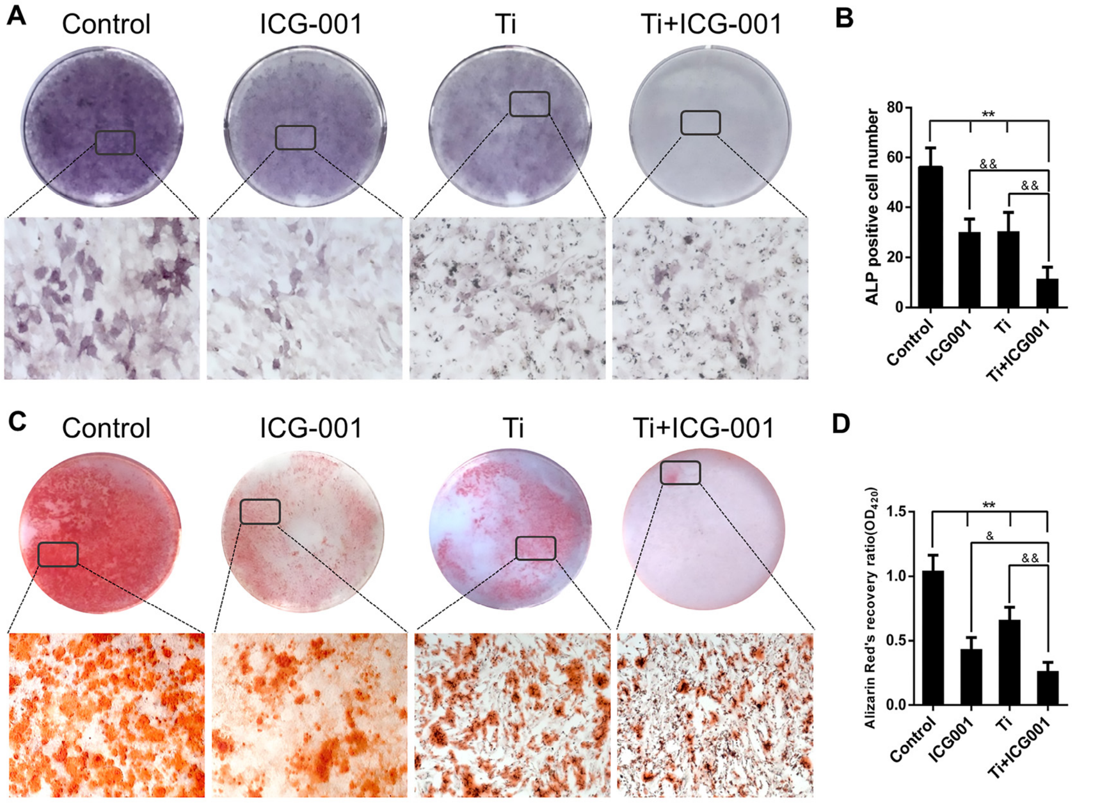

Figure. S2. ICG-001 inhibited osteogenic differentiation and mineralization whether Ti was added or not. (A) ALP staining. (B) The number of ALP-positive cells. (C) ARS staining. (D) Semi-quantitative analysis of ARS staining. Cell differentiation was induced for 7 or 21 days. The concentration of titanium particles and ICG-001 was 5 μg/cm^2 and 20 μM, respectively. Data are presented as means ± SD, n= 9 for ALP, ARS staining and Semi-quantitative analysis of ARS. **P*<0.05 and ***P*<0.01, compared with the control group. &*P*<0.05 and &&*P*<0.01, compared with the Ti+ICG-001 group.
